# Synthesis, Characterization, and Magnetoresistive Properties of the Epitaxial Pd_0.96_Fe_0.04_/VN/Pd_0.92_Fe_0.08_ Superconducting Spin-Valve Heterostructure

**DOI:** 10.3390/nano11010064

**Published:** 2020-12-29

**Authors:** Igor Yanilkin, Wael Mohammed, Amir Gumarov, Airat Kiiamov, Roman Yusupov, Lenar Tagirov

**Affiliations:** 1 Institute of Physics, Kazan Federal University, Kremlyovskaya Str. 18, 420008 Kazan, Russia; yanilkin-igor@yandex.ru (I.Y.); waelmohammed88@yahoo.com (W.M.); amir@gumarov.ru (A.G.); airatphd@gmail.com (A.K.); 2 Department of Physics, Faculty of Science, Minia University, Minia 61519, Egypt; 3 Zavoisky Physical-Technical Institute, FRC Kazan Scientific Centre of RAS, 420029 Kazan, Russia

**Keywords:** spintronics, superconducting spin-valve, epitaxial thin-film heterostructure, palladium-iron alloy, vanadium nitride

## Abstract

A thin-film superconductor(S)/ferromagnet(F) F1/S/F2-type Pd_0.96_Fe_0.04_(20 nm)/VN(30 nm)/Pd_0.92_Fe_0.08_(12 nm) heteroepitaxial structure was synthesized on (001)-oriented single-crystal MgO substrate utilizing a combination of the reactive magnetron sputtering and the molecular-beam epitaxy techniques in ultrahigh vacuum conditions. The reference VN film, Pd_0.96_Fe_0.04_/VN, and VN/Pd_0.92_Fe_0.08_ bilayers were grown in one run with the target sample. In-situ low-energy electron diffraction and ex-situ X-ray diffraction investigations approved that all the Pd_1−x_Fe_x_ and VN layers in the series grew epitaxial in a cube-on-cube mode. Electric resistance measurements demonstrated sharp transitions to the superconducting state with the critical temperature reducing gradually from 7.7 to 5.4 K in the sequence of the VN film, Pd_0.96_Fe_0.04_/VN, VN/Pd_0.92_Fe_0.08_, and Pd_0.96_Fe_0.04_/VN/Pd_0.92_Fe_0.08_ heterostructures due to the superconductor/ferromagnet proximity effect. Transition width increased in the same sequence from 21 to 40 mK. Magnetoresistance studies of the trilayer Pd_0.96_Fe_0.04_/VN/Pd_0.92_Fe_0.08_ sample revealed a superconducting spin-valve effect upon switching between the parallel and antiparallel magnetic configurations, and anomalies associated with the magnetic moment reversals of the ferromagnetic Pd_0.92_Fe_0.08_ and Pd_0.96_Fe_0.04_ alloy layers. The moderate critical temperature suppression and manifestations of superconducting spin-valve properties make this kind of material promising for superconducting spintronics applications.

## 1. Introduction

Approaching the physical limitations of Moore’s law draws attention to functional elements based on principles different from those implemented in semiconductor technology [1,2,3,4,5]. Underlying physics should allow such elements to overcome the constraints inherent to the complementary metal-oxide-semiconductor (CMOS) electronics. Superconducting logic devices are candidates for supercomputer applications due to their high operating frequency and ultralow energy consumption [6,7,8,9,10]. The conventional Josephson junction [11,12], in which the superconducting current flows due to the phase difference between two superconducting electrodes, is the basic element of superconducting circuits such as single-flux quantum logic (SFQ) [13,14], quantum flux parametron [15,16], reciprocal quantum logic [17,18], and adiabatic superconductor logic [19]. Single-quantum logic is the most developed one in application to supercomputing [20,21]; within its framework, weak ferromagnetic couplings in Josephson junctions [22,23,24,25] were proposed as the intrinsic phase shifters of the superconducting wave function. “Magnetic Josephson contacts” have found their application in logic gates [26,27,28,29,30,31] and random access and cache memories [32,33,34,35,36,37], significantly reducing the number of Josephson junctions and interconnects in the circuitries [38] and providing wide operation margin tolerances and low bit-error-rates [39]. The recent proposal of ultralow power artificial synapses utilizing magnetic Josephson junctions with magnetic nanoclusters in the weak link [40] further extends the range of applications towards large-scale neuromorphic computing networks.

The functional Josephson structures mentioned above use niobium as a superconductor, mainly because it is an elemental metal with the superconducting transition temperature *T*_c_(Nb) = 9.25 K, significantly higher than the temperature of liquid helium *T*_LHe_ = 4.2 K at normal conditions, and thin-film niobium technology is well matured. However, niobium films/layers are polycrystalline, and heterostructures with ferromagnetic metals of the iron group and their alloys are not epitaxial. At the same time, some cubic nitrides of transition metals, such as vanadium, niobium, molybdenum, and tantalum, have a *T*_c_ in the range of 8–16 K [41] and lattice constants *a* ≈ 412–439 pm matching the epitaxy conditions with some ferromagnetic metals, their alloys (*a* ≈ 286–352 pm), and magnesium oxide (*a* ≈ 421 pm) as a substrate. They can serve as materials of fully epitaxial core heterostructures for superconducting spintronics. Moreover, superconducting molybdenum nitride MoN_y_ is a material incorporated already in Josephson junction foundries [8,9,42] and used there for interconnects possessing high kinetic inductance. Unlike in semiconductor industries, where the core semiconductor structures of integrated circuits are grown epitaxially ensuring high stability and reproducibility of their characteristics, technologies engaged in niobium foundries were not intended for epitaxial structure synthesis. Therefore, in the presented research, we aimed to study the general possibility of synthesizing fully epitaxial nanoscale-thickness heterostructures for superconducting spintronic circuits.

For multilayer heteroepitaxial superconductor–ferromagnet structures, we chose cubic vanadium nitride (VN) as a superconductor for the following reasons: (1) for VN, the cubic phase is thermodynamically stable [43] (Chapter 11, Section 9.3) (although there are indications of a structural transition to the tetragonal phase below *T*_s_ ~250 K [44] for films with a thickness of 300 nm grown epitaxially on MgO(011) and MgO(001) substrates); (2) the superconducting transition temperature in the bulk, *T*_c_(VN) = 8.2 K [43] (Ch. 11, §9.4) is only a degree lower than that for niobium and also significantly higher than *T*_LHe_ = 4.2 K; and (3) the same symmetry (fcc B1), and the lattice parameter of the bulk VN *a*_0_(VN) = 412.6 pm [43] (Chapter 11, Section 9.4), differing by only 2% from that for magnesium oxide (*a*_0_(MgO) = 421.2 pm). As a ferromagnet, we chose the palladium-iron alloy Pd_1−x_Fe_x_ (x < 0.1) because: (1) it has cubic symmetry (fcc) and long-term stability of properties at low temperatures; (2) the ferromagnetic transition temperature, magnetization, and magnetic hardness can vary over wide ranges by changing the iron content *x* in the alloy [45,46,47,48]; (3) the lattice constant of Pd_1−x_Fe_x_, *a*_0_(PdFe) ≈ 388 pm [48] differs by 6% and 8% from that for VN and MgO, respectively; and (4) magnetic Josephson junction memory was demonstrated [24] utilizing the Pd_0.99_Fe_0.01_ alloy. Cubic symmetry and the closeness of the lattice constants of all three materials create prerequisites for the epitaxial growth of multilayer structures based on VN and Pd_1−x_Fe_x_.

Particular bilayer samples formed by the vanadium nitride and the Pd_1−x_Fe_x_ alloy on MgO(001) were grown in the course of developing the approach to the synthesis of such heterostructures: MgO(001)/VN/Pd_0.96_Fe_0.04_ was studied in [49] with a focus at the magnetic anisotropy of epitaxial Pd_0.96_Fe_0.04_ on VN; MgO(001)/Pd_0.96_Fe_0.04_/VN and MgO(001)/VN/Pd_0.92_Fe_0.08_ bilayers were investigated in [50] with the focus on their electrical properties. In this work, we aimed to synthesize fully epitaxial, three-layer pseudo-spin-valve (spin-valve without exchange bias, but with different coercivities of the functional ferromagnetic layers) heterostructure MgO(001)/Pd_0.96_Fe_0.04_(20 nm)/VN(30 nm)/Pd_0.92_Fe_0.08_(12 nm). Magnetoresistance measurements demonstrate that the structure exhibits superconducting spin-valve effect. The underlying phenomena behind the magnetic Josephson junctions and superconducting spin-valves are the superconductor–ferromagnet proximity effects [51,52,53].

Indeed, at the contact of superconductor (S) and ferromagnet (F), the wave function of the superconducting condensate penetrates into the ferromagnetic metal, oscillates, and decays with a distance from the S/F interface. In a magnetic Josephson heterostructure, the ferromagnetic weak link is sandwiched by two superconductors. If the link accommodates one node of the oscillating pairing wave function, the sandwich sides acquire opposite phases. It is a basic idea realized in the so-called Josephson pi-junctions or pi-shifters [22,23,24,25]. In a heterostructure with two ferromagnetic layers, where the direction of the magnetic moment of the one is chosen as the quantization axis, the oscillation phase depends on mutual orientation of the magnetic moments of the layers. This, in turn, affects the interference of the superconducting pairing wave functions in the layers, and it becomes possible to control the superconductivity in a heterostructure by manipulating the magnetic configurations. Such structures are called superconducting spin-valves (see reviews [51,52] and citations therein).

A more rigorous consideration allows us to assert that three types of superconducting pairings can be realized in thin-film superconductor–ferromagnet heterostructures: singlet, triplet with zero projection of the total spin of the Cooper pair, and triplet with equal spin pairing [53]. The first two take place for collinear configurations of magnetizations, while the last for non-collinear ones. Various designs have been proposed for heterostructures realizing either *T*_c_ -controlled spin-valves or Josephson spin-valves with the critical current control [54], some of which were implemented in experiments (see [9,10,25,55,56,57,58]).

The magnetoresistance measurements have shown that the pseudo-spin-valve heterostructure MgO(001)/Pd_0.96_Fe_0.04_(20 nm)/VN(30 nm)/Pd_0.92_Fe_0.08_(12 nm)/Si exhibits inverse superconducting spin-valve effect.

## 2. Materials and Their Characterization

### 2.1. Sample Synthesis and In Situ Characterization of the Crystallinity and Chemical Composition

We utilized a four-chamber ultra-high-vacuum (UHV) system for the synthesis and analysis of thin-film structures (SPECS+BESTEC, Berlin, Germany). All chambers are connected by an UHV transfer line (with a residual pressure of ~5 × 10^−10^ mbar), which allows to move a sample under synthesis from one chamber to another without breaking UHV conditions.

**Figure 1:**
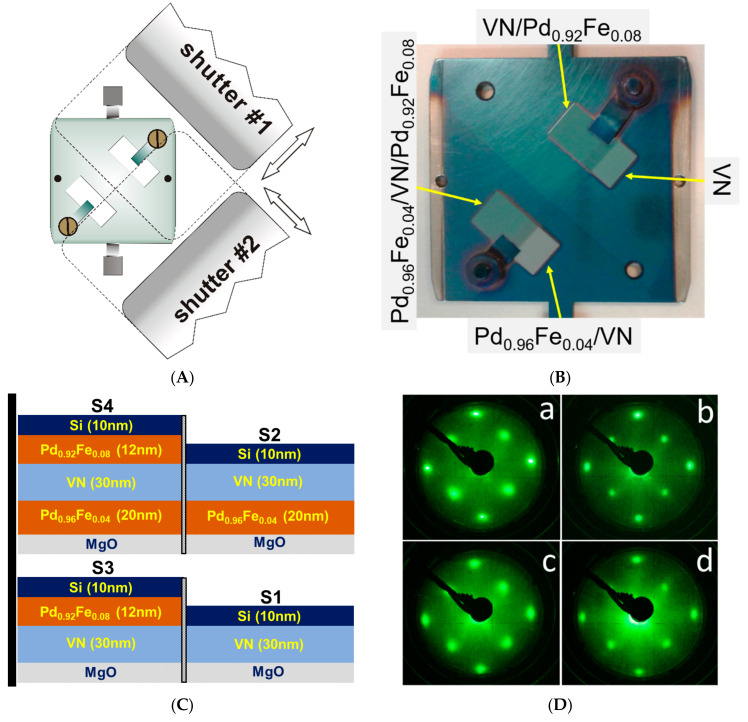
Deposition of the four-sample series in one run: (**A**) layout of the sample holder and shutters; (**B**) photo of the sample holder with the synthesized structures; (**C**) sketch of the sample’s structures; and (**D**) LEED patterns (a, pristine MgO substrate; b, MgO/Pd_0.96_Fe_0.04_ structure; c, MgO/Pd_0.96_Fe_0.04_/VN structure; d, MgO/Pd_0.96_Fe_0.04_/VN/Pd_0.92_Fe_0.08_ structure).

With our approach, we intended to obtain a series of heterostructures with different layer sequences though identical parameters of each particular layer. Its implementation is illustrated by Figure 1. Two single-crystalline epi-polished MgO(001) (referred to as MgO below) substrates from CRYSTAL GmbH, Berlin, Germany, with the size of 10 × 5 × 0.5 mm^3^ were mounted on a molybdenum sample holder with molybdenum clips, as shown in Figure 1A,B. In the vacuum system, they were first annealed for 5 min at 800 °C in a molecular beam epitaxy (MBE) chamber (SPECS) with a residual pressure of ~9 × 10^−11^ mbar. In the same chamber, one of the substrates was obscured with a special sliding shutter #1 (see Figure 1A), and a layer of Pd_1−x_Fe_x_ alloy with a nominal iron content *x* = 0.04 was deposited on the substrate that was left open. Metallic palladium (Pd with a purity of 99.95%, EVOCHEM GmbH, Offenbach, Germany) and iron (Fe with a purity of 99.97%, ChemPur GmbH, Karlsruhe, Germany) were co-evaporated from high-temperature cells (CreaTec GmbH, Erligheim, Germany) calibrated with the quartz microbalance to obtain a desired composition of the Pd_1−x_Fe_x_ alloy. The temperatures of the palladium and the iron cells that provided the elemental composition of Pd/Fe ≈ 96/4, were 1273.0 and 1154.0 °C (±0.1 °C), respectively. The layer thickness of 20 nm was pre-determined by the deposition time and later confirmed ex situ with the Bruker DektakXT stylus profilometer (Bruker Corp., Tucson, AZ, USA) (the height accuracy is ~0.5 nm). Epitaxial growth of the Pd_0.96_Fe_0.04_ layer was achieved using a three-step procedure described in detail in our earlier paper [48].

A layer of the VN compound, common for both substrates, was synthesized by reactive DC magnetron sputtering (MS) in a separate UHV chamber with a base pressure of 8 × 10^–10^ mbar (BESTEC). The metallic vanadium disk of 99.95% purity (LLC GIRMET, Moscow, Russia) was used as a target. The deposition conditions were adapted to our equipment from the works of the Illinois group [59,60]. The substrate had a temperature of 500 °C during this process. A mixture of argon (Ar) and nitrogen (N_2_) with a purity of >99.9999% in the ratio of Ar:N_2_ = 60:40 was fed to the magnetron as the reactive plasma gas. During the deposition, the pressure of the Ar/N_2_ gas mixture in the MS chamber was automatically maintained at 6 × 10^−3^ mbar. With the magnetron power of 50 W and the distance between the target and the substrate of 20 cm, the deposition rate was 0.2 nm/min. The deposited VN layer thickness was 30 ± 0.4 nm. Adjustable shutters of the MS chamber were not used, and the VN layer was uniformly deposited to both substrates using the rotation of the holder around the axis normal to the holder plane.

After transferring the sample holder back to the MBE chamber, the third layer of Pd_1−x_Fe_x_ alloy was synthesized, this time with a nominal composition of Pd_0.92_Fe_0.08_ and a thickness of 12 nm. The last ensured identical magnetic moment per unit area of the Pd_0.96_Fe_0.04_ and Pd_0.92_Fe_0.08_ layers (important for magnetic configurations of the system, see below). Before the deposition, approximately half of the area of both substrates was obscured with shutter #2 (see Figure 1A). The temperatures of the effusion cells were 1273.0 °C for Pd and 1184.0 °C for Fe. As a result of the described synthesis approach, four different structures were formed on the substrates (see Figure 1C) with the top layers of nominally VN (Samples S1 and S2) and Pd_0.92_Fe_0.08_ (Samples S3 and S4).

Upon completion of each deposition step, the crystallinity of the top layer was monitored in situ using the low-energy electron diffraction (LEED) setup built-in to the MBE chamber. The high-contrast pattern in Figure 1Db, obtained from a macroscopic region of the film surface (compare with the LEED pattern of the pristine MgO substrate, Figure 1Da), indicates that the Pd_0.96_Fe_0.04_ film is single-crystalline.

The LEED image from the VN layer is shown in Figure 1Dc, and from the Pd_0.92_Fe_0.08_ layer in Figure 1Dd. Both images display a clear diffraction pattern of cubic symmetry, almost identical to that of the MgO substrate (Figure 1Da) and Pd_0.96_Fe_0.04_ (Figure 1Db). The LEED images allow us to conclude that all the three layers, Pd_0.96_Fe_0.04_, VN, and Pd_0.92_Fe_0.08_, are single-crystalline with the identical orientation of the principal axes in the structure’s plane. This, in turn, indicates that the “cube-on-cube” epitaxial growth mode is realized through all three layers. Thus, Sample S4 represents the desired three-layer, heteroepitaxial structure of Pd_0.96_Fe_0.04_/VN/Pd_0.92_Fe_0.08_ on a single-crystal MgO(001) substrate.

**Figure 2:**
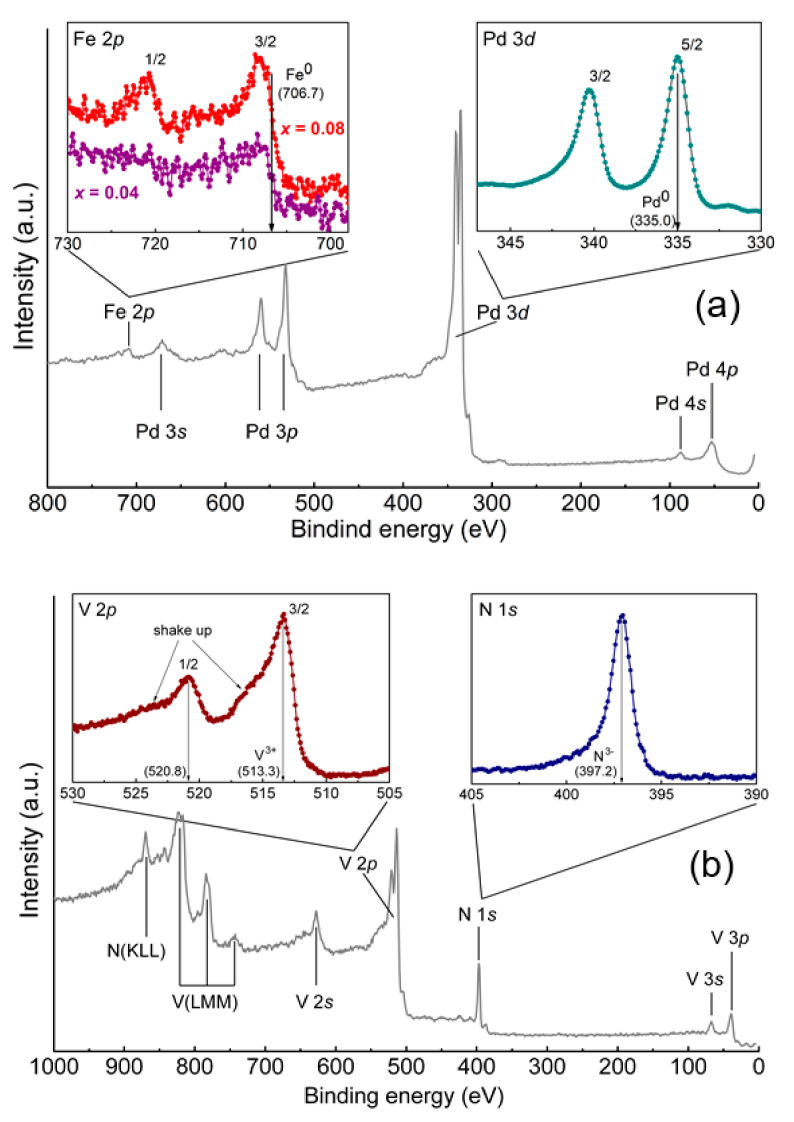
XPS (X-ray photoelectron spectroscopy) spectra of the top layer: (**a**) after depositions of the Pd_1−x_Fe_x_ alloy layer, where the overview spectrum is shown for the Pd_0.92_Fe_0.08_ layer; and (**b**) after deposition of the VN-layer, where callouts show high-resolution spectra in the particular ranges of binding energies of the constituent elements.

The elemental composition of each of the Pd_1−x_Fe_x_ alloy layers was monitored using the UHV X-ray photoelectron spectroscopy (XPS) in the analytical chamber of the vacuum setup (base pressure < 3 × 10^−10^ mbar) equipped with the Mg-K_α_ X-ray source and a hemispherical energy analyzer Phoibos-150 (all from SPECS). Figure 2a shows an overview XPS spectrum of the MgO/VN/Pd_0.92_Fe_0.08_ thin-film heterostructure after deposition; high-resolution spectra of the Fe 2p and Pd 3d states are shown in the insets. The binding energies of the Fe 2p_1/2_, Fe 2p_3/2_, Pd 3d_3/2_, and Pd 3d_5/2_ states are 721.0, 707.7, 340.2, and 335.0 eV, respectively, which correlate well with the literature data [48,61]. Analysis of the XPS spectrum using the CasaXPS software [62] has given the composition of the bottom Pd_1−x_Fe_x_ alloy layer of Pd:Fe = 96.3:3.7 with an accuracy of ~0.5. More details on processing the XPS spectra of Pd_1−x_Fe_x_ alloys can be found in [48].

Analysis of the XPS spectrum of the top Pd_0.92_Fe_0.08_ layer (Sample S4, Figure 1C), performed following the same procedure as for the Pd_0.96_Fe_0.04_ layer, confirmed its elemental composition of Pd:Fe = 92.0:8.0 with an accuracy of ~0.5.

Figure 2b shows the XPS spectra of the VN layer. The binding energies of the V 2p_1/2_, V 2p_3/2_, and N 1s states are 520.8, 513.3, and 397.2 eV, respectively, which are very close to the data reported in the literature for crystalline VN [63]. The presence of a satellite at a binding energy of 515.5 eV is characteristic of the V in the nitride compound [63]. No traces of oxygen or carbon at the surface of the VN layer were found within the sensitivity of the XPS spectrometer (~0.1%). According to the XPS data, the stoichiometry of the layer was V:N = 52.5:47.5 with an accuracy of ~0.5.

Finally, before removing the substrates with four formed samples from the vacuum system, they were capped with 10 nm of high-resistivity silicon in the MS chamber to protect them from the oxidation in air. Nevertheless, in between the ex situ measurements, the samples were stored in a glovebox (MBraun) under a cryogen-pure nitrogen atmosphere. For subsequent measurements, each substrate with the formed samples was cut with a diamond saw into three parts to exclude the transition regions. Only homogeneous samples were used for the measurements. The key object of the studies here was the three-layer MgO/Pd_0.96_Fe_0.04_/VN/Pd_0.92_Fe_0.08_ (S4) sample, while Samples S1–S3 played the role of references to identify the contribution of each layer to the magnetic configurations and magnetoresistance features of Sample S4.

### 2.2. X-ray Diffraction Analysis

**Figure 3:**
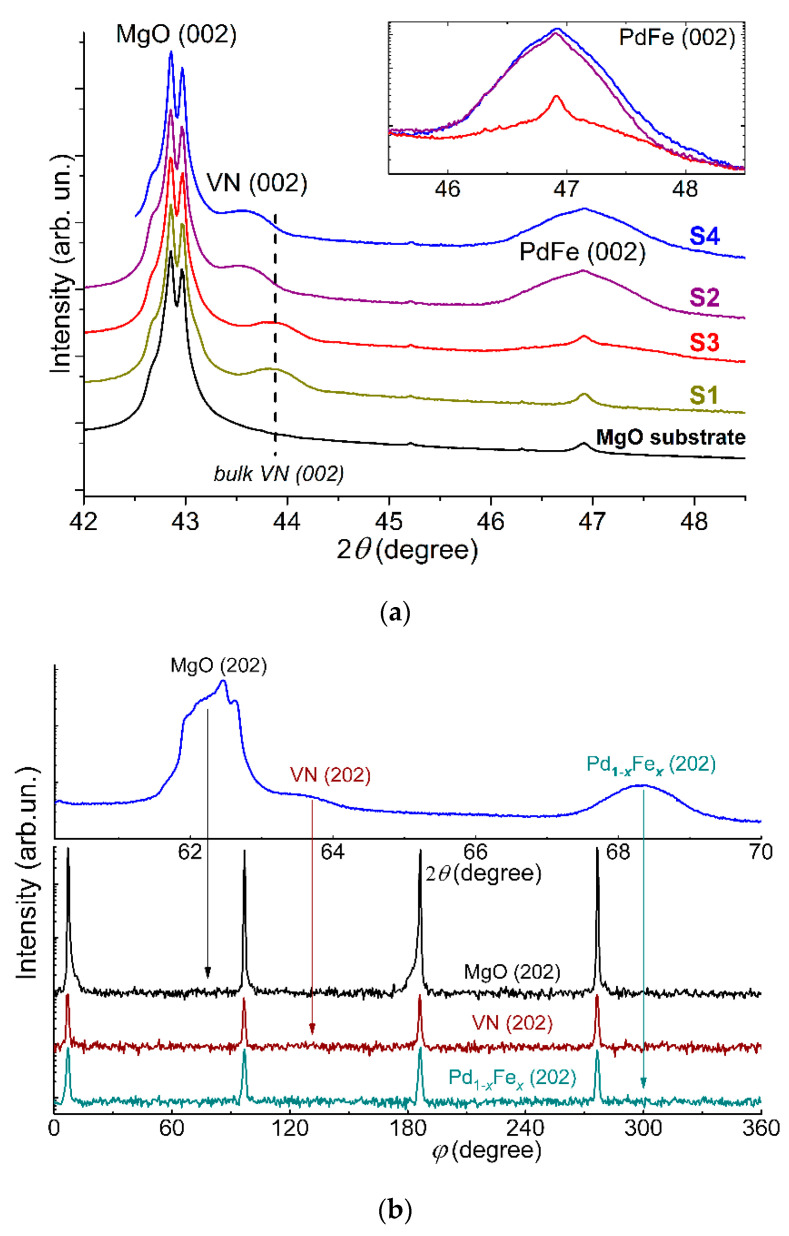
(**a**) X-ray diffractogram (*θ*-scan) of Samples S1-S4 and the pristine MgO(001) substrate, recorded before the thin film deposition (see legends). (**b**) X-ray diffraction *φ*-scans of Sample S4; the Eulerian cradle angle *χ* was set to 45° to detect the <220> XRD-maxima. The *θ*-angle values at which the *φ*-scans were recorded for each material are indicated by vertical arrows.

In situ LEED results were confirmed by ex situ X-ray diffraction (XRD, BRUKER D8, Bruker AXS GmbH, Karlsruhe, Germany) utilizing Cu-K_α_ radiation (λ = 1.5418 Å) in the Bragg–Brentano geometry in the range of 2*θ* angles from 17° to 82° with the step of 0.0153°. XRD patterns at room temperature of the pristine MgO(001) substrate and Samples S1 (MgO/VN/Si), S2 (MgO/Pd_0.96_Fe_0.04_/VN/Si), S3 (MgO/VN/Pd_0.92_Fe_0.08_/Si), and S4 (MgO/Pd_0.96_Fe_0.04_/VN/Pd_0.92_Fe_0.08_/Si) are shown in Figure 3a. The *θ*–2*θ* scans indicate the single-crystalline structure of the layers in the studied samples. In the figure, one can identify the (002) maxima from the MgO substrate, VN (30 nm) layer, and Pd_0.96_Fe_0.04_ (20 nm) and/or Pd_0.92_Fe_0.08_ (12 nm) layer(s).

The figure shows a significant relative shift of the (002) XRD-maxima from the VN layer of Samples S1 and S3 with respect to Samples S2 and S4, clearly correlating with the material that served as a substrate for the VN layer. For Samples S1 and S3, MgO (a = 421.2 pm) was the substrate for the VN (a = 412.6 pm). Therefore, the lattice of the VN film is stretched a bit in its plane; accordingly, the interplanar distance along the normal is reduced, and the angle of the (002) maximum is increased. For Samples S2 and S4, the Pd_0.96_Fe_0.04_ (a = 388 pm) served as the substrate for the VN. The VN lattice is then compressed in its plane; accordingly, the interplanar distance along the normal is increased, and the angle of the (002) maximum is reduced.

For the (002) maximum of the Pd_1−x_Fe_x_, the quantitative difference in its angular position is visible only at the instrumental level. First, in contrast to the VN case, both “substrates” for the growth of Pd_1−x_Fe_x_ layers cause the tensile strain; as a result, the maximum shifts towards larger angles though the shift magnitudes differ slightly. Second, the maxima from the Pd_0.96_Fe_0.04_ and Pd_0.92_Fe_0.08_ layers overlap, thus deposition to different “substrates” mainly leads to the broadening of the integral peak.

X-ray diffraction *φ*-scans are shown in Figure 3b. The Eulerian cradle angle *χ* was set to 45° to detect the <220> XRD-maxima. The *φ*-scans for both layers, VN and Pd_1−x_Fe_x_, clearly show cubic single-crystalline symmetry of the deposited films with the coinciding directions of the principal axes. For comparison, the *φ*-scan of the pristine single-crystal MgO(001) substrate recorded in the same geometry is shown in Figure 3b.

A significant broadening of the diffraction maxima of VN and Pd_1−x_Fe_x_ is primarily associated with a small coherent scattering range τ along the normal to the film plane (Scherrer broadening). The XRD data taking into account the instrumental function [48] lead to the estimates of *τ* ≈ 22.0 nm for Pd_0.96_Fe_0.04_, *τ* ≈ 12.6 nm for Pd_0.92_Fe_0.08_, and *τ* ≈ 30.4 nm for VN, which quantitatively agree with the thicknesses determined from the calibrated quartz microbalance. Thus, the LEED and XRD datasets indicate that thin films of Pd_1−x_Fe_x_ and VN in the MgO/Pd_0.96_Fe_0.04_/VN/Pd_0.92_Fe_0.08_ heterostructure grew in a single-crystal epitaxial cube-on-cube mode.

## 3. Results

### 3.1. Magnetic Properties

**Figure 4:**
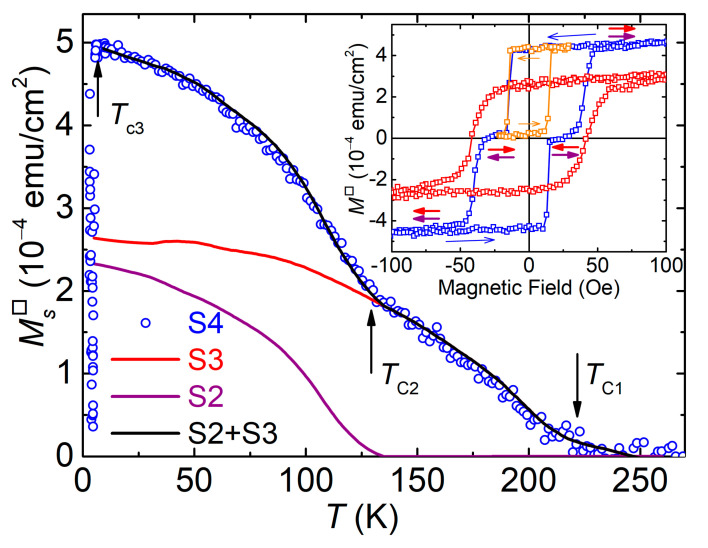
The saturation magnetic moment per unit area as a function of temperature for the pseudo-spin-valve MgO/Pd_0.96_Fe_0.04_/VN/Pd_0.92_Fe_0.08_ heterostructure S4 measured with an applied magnetic field of 500 Oe. The black solid line is a sum of the magnetic moments of Samples S2 and S3 in the temperature range above the superconducting transition. The inset shows magnetic hysteresis loops for the same structure measured at 6 K: blue squares, major hysteresis loop of the S4 sample; orange squares, minor hysteresis loop of the S4 sample; red squares, hysteresis loop of the S3 sample. Pairwise purple and red arrows depict magnetic moment directions of the Pd_0.92_Fe_0.08_ and Pd_0.96_Fe_0.04_ layers, respectively. The ferromagnetic transition temperatures are *T_C_*_1_ ≈ 220 K for Sample S3 and *T_C_*_2_ ≈ 127 K for Sample S2, while the superconducting transition temperature *T_c_*_3_ = 5.42 K.

The results of the magnetic moment measurements of the hetero-epitaxial pseudo-spin-valve structure MgO/Pd_0.96_Fe_0.04_/VN/Pd_0.92_Fe_0.08_/Si (S4) are shown in Figure 4. The dependence of *M*_S4_(*T*) (blue open circles) is well reproduced by the sum of *M*_S2_(*T*) and *M*_S3_(*T*) measured on the reference samples of Series S2 and S3 (purple and red lines in Figure 4). The dependence of the saturation magnetic moment on temperature clearly reveals three critical regions corresponding to the Curie temperatures for ferromagnetic layers (see vertical arrows in the figure) and the onset of the superconductivity at 5.42 K, which manifests itself through the Meissner effect.

The major magnetic hysteresis loop of the pseudo-spin-valve Sample S4 is shown in the inset of Figure 4 (blue open squares); it has a stepped shape since the Pd_0.96_Fe_0.04_ and Pd_0.92_Fe_0.08_ layers have different coercive fields, ~15 and ~40 Oe, respectively. The magnetic field was applied along the easy in-plane magnetization axis. The field sweep started from the saturated state in the positive field corresponding to the parallel (P) alignment of the Pd_0.96_Fe_0.04_ and Pd_0.92_Fe_0.08_ layer magnetic moments. Measurements of the magnetic hysteresis loop of Sample S3 (MgO(001)/VN/Pd_0.92_Fe_0.08_/Si), red symbols in the inset, show that the Pd_0.92_Fe_0.08_ layer has a higher coercive field. In the range of the antiparallel (AP) alignment of the Pd_0.96_Fe_0.04_ and Pd_0.92_Fe_0.08_ layer moments, the total magnetization of the sample is close to zero, which was intentionally pre-determined by choosing their appropriate thicknesses (20 and 12 nm, respectively) based on the iron concentration dependence of Pd_1−x_Fe_x_ alloys magnetization [47]. The orange symbols in the inset show a minor hysteresis loop recorded starting from the saturated state in the positive field direction. The minor loop corresponds to the switching between the P and AP magnetic configurations.

### 3.2. Superconducting Properties and Magnetoresistance

**Figure 5:**
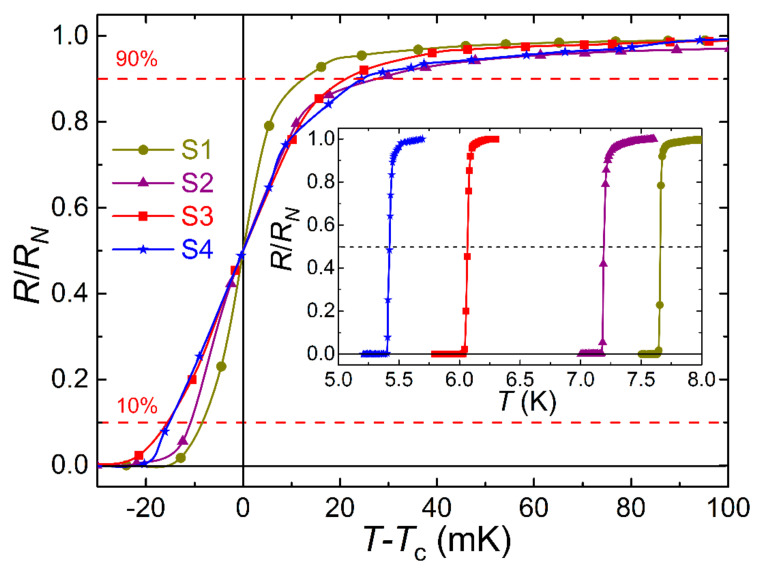
Resistive superconducting transitions of the single VN film (S1), the bilayer Structures S2 and S3, and the pseudo-spin-valve Structure S4, at zero magnetic field. Lines connecting symbols are drawn using a modified Bezier algorithm. Measurements with the sensing currents of 20 and 100 μA did not reveal any difference in the width and shape of the transitions.

The superconducting transition temperature was determined from the resistance dependence on temperature in the range of 4.2–300 K. A four-probe resistance measurement scheme was used (Quantum Design PPMS-9). Figure 5 shows the superconducting transitions for Samples S1–S4 with the abscissa rescaled to track the evolution of the transition width. The raw *R*(*T*) transitions are shown in the inset to the figure. Table 1 contains the data on the residual resistance ratio *RRR* (the ratio of the resistance *R*_300K_ at room temperature to the resistance *R*_10K_ at 10 K), the superconducting transition temperature *T*_c_ (according to the criterion of the middle of the transition), and the width of the superconducting transition according to the criterion of 10–90. The structural quality of our 30 nm thick VN film, manifested in *RRR* ~5.1 >> 1 and low resistivity at room temperature ~42.5 μΩ·cm [50], is one of the best [41,44,64,65,66].

The most notable feature of the *T*_c_ measurement results is a small width of the superconducting transition of the MgO/Pd_0.96_Fe_0.04_(20 nm)/VN(30 nm)/Pd_0.92_Fe_0.08_(12 nm)/Si (S4) sample of about 40 mK according to the 10–90% criterion, and the absence of “tail” of the resistive transition towards low temperatures.

**Figure 6:**
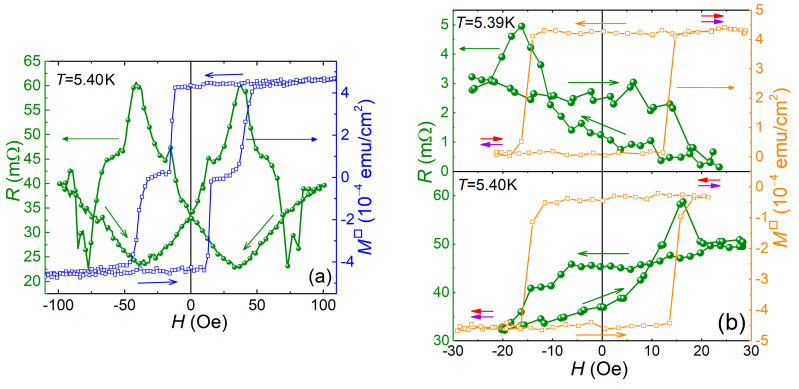
Dependence of electric resistance of the tri-layer MgO/Pd_0.96_Fe_0.04_/VN/Pd_0.92_Fe_0.08_/Si (S4) pseudo-spin-valve sample on the magnetic field: (**a**) remagnetization along the major magnetic hysteresis loop; and (**b**) remagnetization along the minor magnetic hysteresis loops.

The magnetoresistance *R*_S4_(*H*) of the pseudo-spin-valve Sample S4 was measured in the same geometry as the magnetic hysteresis, i.e., starting from the P magnetic configuration and the field applied in-plane along the easy magnetization axis. The measurement temperature was set to 5.40 K. To achieve reproducible *R*(*H*) results, the temperature of the sample had to settle for at least 1 h. Figure 6a shows the measurement results by scanning the magnetic field over the major hysteresis loop. The observed dependence is symmetric with respect to the origin. With the decrease of the magnetic field from, e.g., +100 Oe, resistance first drops, reaches a minimum at ~30 Oe, and then rises. After passing zero field, it rises further and, after about −30 Oe, drops again. Scanning the field in an opposite direction produces symmetric about *H* = 0 Oe curve. The *R*_S4_(*H*) dependence exhibits peak-like anomalies that obviously correlate with the magnetization reversals of the Pd_0.96_Fe_0.04_ and Pd_0.92_Fe_0.08_ ferromagnetic layers. The dip pits at the field values about ±80 Oe do not correlate with any changes in magnetic configurations in these field ranges. Magnetoresistance measurements of the MgO/VN/Si (S1) reference sample (Appendix A) which was obtained in the same deposition process with Samples S2–S4, showed that the dips in magnetoresistance originate from the VN film itself.

To better understand the influence of the magnetic configurations on superconductivity in the S4 pseudo-spin-valve structure, we investigated its magnetoresistance with the field sweeping within the minor hysteresis loop (Figure 6b). The top panel shows the magnetic field sweep causing the switching from the P state of the ferromagnetic Pd_1−*x*_Fe*_x_* layers in the positive field direction to the AP state occurring at *H* ≈ −24 Oe, and back at *H* ≈ +28 Oe to complete the minor loop. The bottom panel of Figure 6b shows a similar measurement of the *R*(*H*) dependence within the minor loop, starting from the P-configuration at negative fields. Regardless of the history, the resistance of the structure is higher with the AP configuration of magnetic moments than with the P configuration. Thus, an inverse spin-valve effect is observed.

## 4. Discussion

A decrease in the superconducting transition temperature of the VN compound film in a heterostructure with a ferromagnetic alloy Pd_1−x_Fe_x_ is a manifestation of the inverse superconductor/ferromagnet (S/F) proximity effect (see review [51] and references therein). Recent predecessors for the MgO/Pd_0.96_Fe_0.04_/VN/Pd_0.92_Fe_0.08_/Si (S4) heterostructure studied in this work were the F1/S/F2 and F1/F2/S spin-valves considered in [67,68], respectively. The calculation for the F1/S/F2 structure [67] predicts a monotonic change in *T*_c_, and hence in the resistance *R*(*H*) at the superconducting transition (Figure 6a), upon transition from the P-configuration (lower *T*_c_) to the AP-one (higher *T*_c_). It is called the direct spin-valve effect. In our case, a significant bump in resistance (decrease in *T*_c_) is observed with a maximum at ~±40 Oe upon magnetization reversal of the Pd_0.92_Fe_0.08_ layer. Although this does not agree with the prediction in [67], it was assumed there that magnetic moments of ferromagnetic layers rotate coherently, keeping the magnetization within the layer uniform. If a non-uniform magnetization state develops in the course of magnetization reversal in any of the two ferromagnetic layers (e.g., following the “exchange spring”/spiral magnets scenario [69,70,71] or remagnetization through the domain intermediate state [72,73]), the triplet superconductivity component emerges that additionally suppresses the *T*_c_ (increases the resistance). For the simplest case of two non-collinearly magnetized layers, this suppression is demonstrated in [68] and coined as triplet spin-valve regime. In our case, the pronounced peaks at about ±40 Oe can be attributed to the development of the triplet pairing in the Pd_0.92_Fe_0.08_ layer. The influence of the Pd_0.96_Fe_0.04_ layer reversal reveals itself in *R*(*H*) data presented in Figure 6b. The measurements showed that a slight inhomogeneity can also appear upon magnetization reversal of the Pd_0.96_Fe_0.04_ layer (resistance spikes at ±55 Oe); however, regardless of the history, the structure resistance is higher when the sample is in the AP state, i.e., we observe an ***inverse*** spin-valve effect (*T*_c_(AP) < *T*_c_(P)).

## 5. Conclusions

A thin-film F1/S/F2-type superconductor/ferromagnet MgO(001)/Pd_0.96_Fe_0.04_/VN/Pd_0.92_Fe_0.08_ heterostructure was synthesized in one run with the reference MgO/VN, MgO/Pd_0.96_Fe_0.04_/VN, and MgO/VN/Pd_0.92_Fe_0.08_ samples in UHV conditions using a combination of molecular beam epitaxy and magnetron sputtering. Structural characterizations showed that the target trilayer heterostructure grew fully epitaxial. Electric resistance measurements have shown sharp superconducting transitions for all four samples without substantial transition broadening upon complication of the sample structure from the single VN layer to the Pd_0.96_Fe_0.04_/VN/Pd_0.92_Fe_0.08_ pseudo-spin-valve trilayer. The pronounced magnetoresistance features observed upon remagnetization of the F1/S/F2 pseudo-spin-valve trilayer in the film plane give evidence of the inverse superconducting spin-valve effect. Such a manifestation of the superconductor–ferromagnet proximity effects paves the way to magnetic Josephson junctions based on fully epitaxial superconductor–ferromagnet heterostructures.

## Figures and Tables

**Table 1:** Electrical and superconducting properties of heteroepitaxial Structures S1–S4 on MgO(001).

Structure	*RRR*	*T*_c_ (K)	Δ*T*_c_ “10–90”(mK)
MgO/VN(30 nm)/Si (S1)	5.13	7.66	21
MgO/Pd_0.96_Fe_0.04_(20 nm)/VN(30 nm)/Si (S2)	3.51	7.20	39
MgO/VN(30 nm)/Pd_0.92_Fe_0.08_(12 nm)/Si (S3)	2.60	6.07	37
MgO/Pd_0.96_Fe_0.04_(20 nm)/VN(30 nm)/Pd_0.92_Fe_0.08_(12 nm)/Si (S4)	2.46	5.41	40

**Figure A1:**
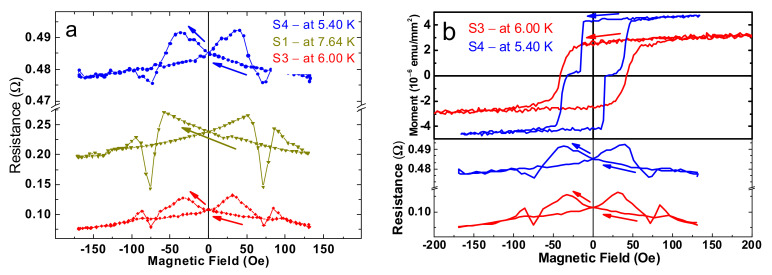
(**a**) *R*(*H*) dependences of the single VN film (S1), the heterostructure VN/Pd_0.92_Fe_0.08_ (S3), and the pseudo-spin-valve structure Pd_0.96_Fe_0.04_/VN/Pd_0.92_Fe_0.08_ (S4). (**b**) Magnetoresistance data for Samples S3 and S4 are shown in the lower panel in conjunction with the magnetic hysteresis loops in the upper panel for the same samples measured in identical geometries of the experiments.

## Data Availability

The data presented in this study are available on request from the corresponding author.

## Appendix A

*R*(*H*) “fast” scans were intended to clarify the origin of pits at the magnetic field values of about ±80 Oe. In this series of quick measurements, the procedure of thermalization, described above, was deliberately shortened to obtain mostly qualitative information. Due to the fast scan, some low-amplitude features of *R*_S4_(*H*) were smeared or lost compared with those in Figure 6. The measurements of the single VN film (Sample S1), the middle scan in Figure A1a, clearly demonstrate that ±80 Oe pits originate from the VN layer.
